# Invasion of *Aedes albopictus* (Diptera: Culicidae) into central Africa: what consequences for emerging diseases?

**DOI:** 10.1186/s13071-015-0808-3

**Published:** 2015-03-31

**Authors:** Carine Ngoagouni, Basile Kamgang, Emmanuel Nakouné, Chistophe Paupy, Mirdad Kazanji

**Affiliations:** Institut Pasteur de Bangui, Bangui, Central African Republic; Centre International de Recherches Médicales de Franceville (CIRMF), BP 769 Franceville, Gabon; Laboratoire des Maladies Infectieuses et Vecteurs: Ecologie, Génétique, Evolution et Contrôle, UMR 224-5290, CNRS-IRD-UM1-UM2, IRD, Montpellier, France

**Keywords:** *Aedes albopictus*, *Aedes aegypti*, Invasive species, Arboviruses, Public health, Central Africa

## Abstract

*Aedes albopictus*, a mosquito native to Asia, has invaded all five continents during the past three decades. It was reported in central Africa in the 2000s, first in Cameroon, and, since then, has colonised almost all countries of the region. The species, originally considered a secondary vector of dengue viruses, has been showed to play a major role in transmission of chikungunya virus in numerous countries, including in the central African region. We review the current spread of *Ae. albopictus* in central Africa, its larval ecology and its impact on indigenous species such as *Ae. aegypti*. We explore the potential of *Ae. albopictus* to affect the epidemiology of emerging or re-emerging arboviruses and discuss the conventional means for its control, while emphasizing the importance of data on its susceptibility to insecticides to cope with potential outbreaks.

## Introduction

During the past three decades, *Aedes albopictus* (Skuse, 1894), an invasive species originating in Asia, has invaded the Americas, Europe and Africa [1,2]. This rapid global spread was favoured by international trade, especially of used tyres [3], and by its physio-local and ecological plasticity, which allow the species to thrive in a wide range of climates and habitats [4]. *Ae. albopictus* is considered to be a vector or potential vector of several pathogens of human and veterinary importance. Viral isolation and vector competence studies have shown that this mosquito is an efficient vector of more than 20 arboviruses [2,5].

In continental Africa, *Ae. albopictus* was first identified in South Africa in 1989, probably due to trade in used tyres from Japan, and it was promptly controlled [6]. It was identified in Nigeria 2 years later as an invasive species [7]. In central Africa, it was first reported in 2000 in Cameroon [8] and has since been found in almost all countries of the region. *Ae. albopictus* is often found with resident species in the same city and larval breeding sites, particularly with *Ae. aegypti* [4,9,10]. Several arboviruses have been isolated from mosquitoes and human samples in central Africa [11-14], but no massive outbreak has been reported before introduction of the new competent vector *Ae. albopictus*. In this paper, we report the current understanding of the biology, behaviour and vector status of this species and discuss the possible role for emerging new arboviruses in central Africa.

## Review

### Current distribution of *Ae. albopictus*

**Figure 1:**
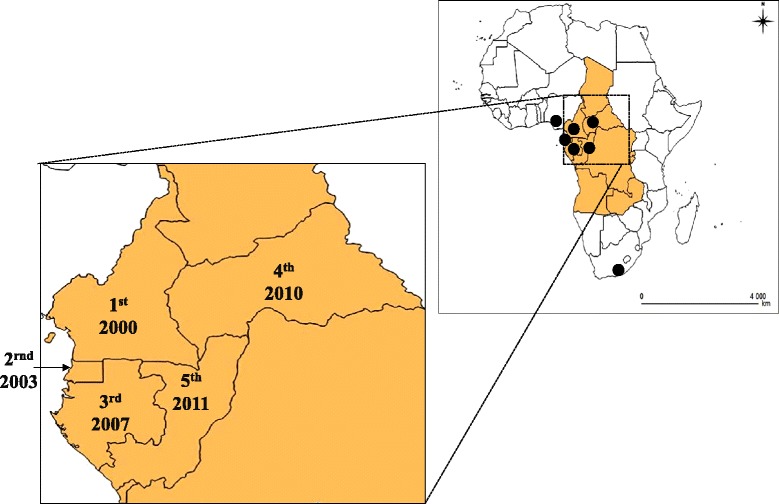
**Chronology of invasion by** ***Ae. albopictus*** **in central Africa.** The black circle represent the continental African countries infested by *Ae. albopictus.*1st: Cameroon in 2000; 2ed: Equatorial Guinea in 2003; 3rd: Gabon in 2007; 4th: Central African Republic in 2010; 5th: Republic of Congo in 2011.

The global spread of *Ae. albopictus* is due mainly to human activities, such as increase in intercontinental trade, especially in the past three decades [5]. In central Africa, *Ae. albopictus* was first described in 2000 in Cameroon [8], then in 2003 in Equatorial Guinea [15], in 2007 in Gabon [16], in 2009 in the Central African Republic (CAR) [17] and in 2011 in Republic of Congo [18] (Figure 1). In Cameroon, entomological investigation on a macro-geographical scale revealed that *Ae. albopictus* is present only in the southern part of the country, which is characterized by an equatorial climate, whereas the native species *Ae. aegypti* is present throughout the country [9,10]. A study in CAR showed that *Ae. albopictus* predominated over *Ae. aegypti* at all sites where both species were sympatric [4], and data on the spatial distribution of *Ae. albopictus* showed that this invasive species is widespread in southern sites, such as Mbaïki, Batalimo, Mongoumba, Boda and Berberati, except in Bouar (located near Cameroon at 6°N latitude), where *Ae. aegypti* is found alone. High densities of *Ae. albopictus* were also reported in several cities in Gabon (Libreville, Lastouville in the south-east, Franceville, Oyem and Cocobeach in the north-west) [19-21]. These observations are consistent with the hypothesis that invasive species are more likely to establish themselves in environments that are similar to their native environment but can also evolve to adapt better to their new environment [22]. The absence of *Ae. albopictus* above 6°N in Africa suggests a climatic limitation for invasion of the species [9,10].

### Biology

**Figure 2:**
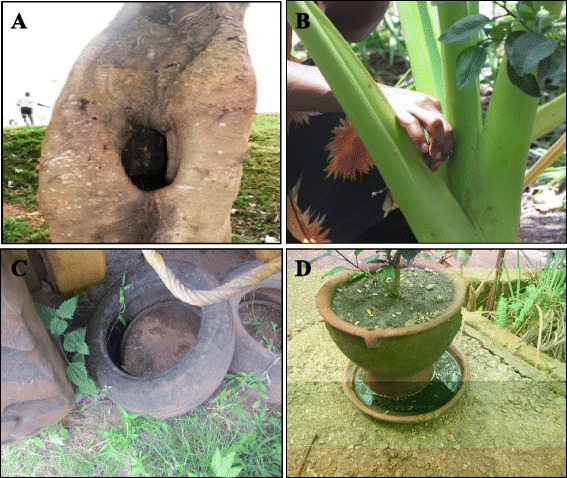
**Examples of larval breeding sites of** ***Ae. albopictus.*** **A**. tree holes; **B**. leaf axil; **C**. used tyres; **D**. flower-pot saucer.

i.) Breeding sites of *Ae. albopictus*
  *Aedes albopictus* has strong ecological plasticity, which allows its rapid adaptation to a wide range of habitats. Studies in central Africa show that its larval breeding sites are diverse, ranging from natural sites (e.g. tree holes, snail shells, rock holes, cacao shells, coconut shells and leaf axils) to artificial containers (e.g. water storage containers, used tyres, tin cans, car wrecks, flower-pots) [4,8-10] (Figure 2). Detailed characterisation of larval ecology in Cameroon and CAR showed that *Ae. albopictus* breeds mainly in used tyres, discarded tanks and flower-pots and prefers containers with plant debris and/or surrounded by vegetation. The most productive containers were used tyres, follow by discarded tanks [4,10].
ii.) Feeding hosts and daily dynamics of host-seeking activity
  *Aedes albopictus* has long been considered mainly zoophilic and able to feed on most groups of cold- and warm-blooded vertebrates, including reptiles, birds and amphibians [2,23,24]. Analysis of ingested blood in outdoor-resting females in Cameroon showed that *Ae. albopictus* preferentially fed on humans rather than on domestic animals (95% of blood meals contained human blood) [25]. These results conflict with the assumption that *Ae. albopictus* is mainly zoophilic [2,23,26] and are consistent with observations made in regions outside Africa, such as Thailand [27], the USA [28], Italy [29] and La Réunion [30]. These results indicate that the authors chose sites where animals were available, while *Ae. albopictus* prefers to feed on humans. The propensity of *Ae. albopictus* females to feed on humans in urban areas in Cameroon is a concern, as it suggests a risk for human–human pathogen transmission. Moreover, observation of a few blood meals in pigs and reptiles, and especially mixed animal–human meals, confirms that this species could act as a bridge vector for zoonotic pathogens [25]. Mosquito collection with a double-net device in Cameroon demonstrated that *Ae. albopictus* females feed during daytime, from 05:00 to 19:00, with a peak from 15:00–19:00 [25]. Although *Ae. albopictus* is sometimes observed indoors, it is generally considered exophilic and exophagic in Africa and elsewhere [5,23,30].

### Interaction with indigenous species *Ae. aegypti*

Numerous studies on the spatial coexistence of *Ae. aegypti* and *Ae. albopictus* have been conducted outside Africa, where the two species are sympatric [31,32]. In North America [33] and Brazil [31], the two species have similar larval ecological niches and often share the same larval habitat. Likewise, in Mayotte, *Ae. albopictus* co-exists with *Ae. aegypti* in 40% of larval habitats [34]. As suggested by Paupy et al. [5], however, the apparent coexistence of the two species could be a transient situation, followed by a reduction [35-37] or displacement [38,39] of the resident species; interspecific larval competition for resources is the most likely reason for this process.

In central Africa, when *Ae. albopictus* was widespread, it was suspected to have played a major role in transmission of the viruses of dengue and chikungunya. Most of the studies therefore focused on viral detection or isolation, and few studies have been conducted on its interactions with the resident species *Ae. aegypti*. Nevertheless, two studies conducted in Cameroon [10] and CAR [4] provide more detail (such as building density, type of container, vegetation around the container and plant debris inside the container) on the spatial distribution and interactions between the invasive species *Ae. albopictus* and the resident species *Ae. aegypti*. Data obtained showed that immature stages of both species colonized a variety of artificial natural breeding sites and were often found together at the same larval development sites. *Ae. albopictus*, however, colonizes preferentially containers containing plant debris or surrounded by vegetation. Thus, although the two vectors are sympatric, significant differences in their relative proportions and their spatial distribution are likely, due to environmental factors (e.g. climate, vegetation and building density). In the detailed study in Bangui (CAR), *Ae. aegypti* species represented the majority in the early rainy season, whereas *Ae. albopictus* was most abundant in the late rainy season. This is probably due to the better tolerance of *Ae. aegypti* eggs to desiccation than those of *Ae. albopictus*, as suggested by Juliano et al. [40]. All the studies undertaken in the sympatric area in central Africa suggest that *Ae. albopictus* tends to supplant the resident species *Ae. aegypti* [4,10,20,41].

### Population genetics and phylogeography

**Figure 3:**
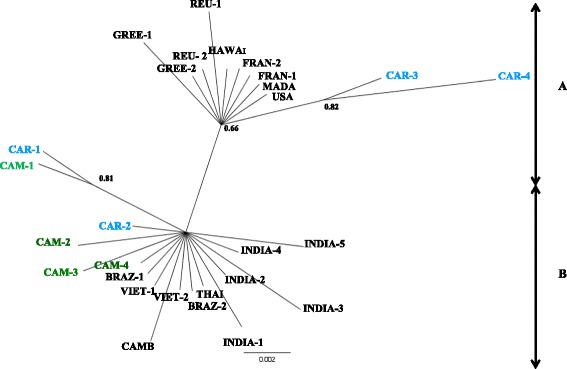
**Phylogeographical tree of** ***Ae. albopictus*** **from CAR and Cameroon based on COI. A**. Subtropical or temperate region: GREE, Greece; REU, Reunion; HAWAI, Hawai; FRAN, France; MADA, Madagascar; USA, United State of the America. **B**. Tropical region: CAR, Central African Republic; CAM, Cameroon; BRAZ, Brazil; VIET, Vietnam; THAI, Thailande; INDIA, India; CAMB, Cambodge.

Since introduction of *Ae. albopictus* into central Africa, genetic studies have been conducted only in Cameroon [42,43] and CAR [4]. Analyses of Cameroonian samples with microsatellite markers showed moderate, statistically significant overall genetic differentiation between samples. No obvious relation between genetic and geographical distances was found, suggesting that the genetic structure has been shaped by additional biotic or abiotic factors. Analysis of mtDNA sequences revealed four haplotypes each for the *COI* and *ND5* genes, with a dominant haplotype shared by all Cameroonian samples [42]. Phylogeographical analysis based on *COI* polymorphism indicated that *Ae. albopictus* populations in Cameroon are related to tropical rather than temperate or subtropical outgroups [42]. Similar analysis of the CAR samples also showed little overall mtDNA diversity [4], which is consistent with the recent introduction of a few founder females or may be related to ubiquitous *Wolbachia* infection in populations of this species, as suggested by Armbruster et al. [44]. Phylogeographical analysis based on *COI* polymorphism indicated that the *Ae. albopictus* haplotype in the CAR population segregated into two lineages (Figure 3), suggesting multiple sources [4]. The moderate genetic diversity observed among Cameroonian and CAR *Ae. albopictus* isolates is in keeping with recent introduction and spread in these countries.

### Impact on health

Invasive mosquito species are defined by their ability to colonize new territories and can affect human health by concurrently harbouring novel pathogens, transmitting native pathogens or transmitting novel pathogens introduced independently [39]. Changes in the epidemiology of arboviruses after the introduction of invasive species have been seen throughout the world, including simultaneous introduction of *Ae. aegypti* and yellow fever virus in the Americas between the 16th and 17th centuries [38], the re-emergence of dengue in Asia after introduction of *Ae. aegypti* [45] and the emergence of dengue in Hawaii after *Ae. albopictus* was established in 2001 [46].

The introduction of *Ae. albopictus* and its subsequent rapid spread in numerous countries of central Africa is particularly disturbing, as it is suspected to have played a major role in the transmission of Chikungunya virus (CHIKV) in Cameroon in 2006 [47] and was the main vector of CHIKV and dengue virus (DENV) in Gabon in 2007 [20,21,41,48]. In Cameroon, additional *Ae. albopictus* populations were shown to be orally susceptible to DENV-2 and highly competent for CHIKV [41]. In Republic of Congo more recently, Mombouli et al. [49] confirmed that *Ae. albopictus* together with the native species *Ae. aegypti* played a role in the dissemination and spread of CHIKV during the 2011 outbreak, after 39 years of absence. The role of *Ae. albopictus* in the transmission of DENV and CHIKV has been recognized since 2009 [5], and, in 2013, Grard et al. [50] provided the first direct evidence of human Zika virus (ZIKV) infection in the Asian tiger mosquito, *Ae. albopictus*, in Gabon. Phylogenetic analysis placed the Gabonese ZIKV at a basic position in the African lineage, in agreement with previously obtained complete sequences of ZIKV strains, indicating an African lineage and an Asian lineage [51]. Therefore, the emergence of ZIKV in Gabon was not due to an imported strain but rather to the diversification and spread of an ancestral strain belonging to the African lineage. These data from Libreville in 2007 are the first proof of human ZIKV infection in an urban environment during concurrent CHIKV and DENV outbreaks and its first occurrence in the invasive mosquito *Ae. albopictus*.

The introduction in central Africa of a new vector that is now known to be competent for more than 20 arboviruses is a public health problem, because three arboviruses (CHIKV, DENV and ZIKV) that are endemic in the region have re-emerged. *Ae. albopictus* can also transmit filarial nematodes, which are primarily parasites of dogs but can also affect humans. Evidence of its transmission by Italian *Ae. albopictus* populations [52,53] has been linked to an increased prevalence of human dirofilariosis [54]. The emergence of a new strain of CHIKV in Gabon shows that *Ae. albopictus* can interfere with the indigenous virus-vector system and augment viral emergence. This is a particular problem in areas such as central Africa where malaria is still a public health problem because of the diversity of pathogens transmitted by mosquitoes. In CAR, where a high infestation index of *Ae. albopictus* has been reported, there is thus an imminent risk for large outbreaks of arboviral infections, such as dengue, chikungunya and zika, as observed elsewhere in the region. It would be interesting to evaluate the vector competence of numerous arbovirus for *Ae. albopictus* populations and *Ae. aegypti* in central Africa to assess the risk for emergence or re-emergence in the region.

### Control of *Ae. albopictus*

In view of the occurrence in central Africa of large outbreaks of dengue and chikungunya, the main diseases transmitted by *Ae. albopictus*, preventive measures are required in all countries of the region, because there is no vaccine or specific treatment against these diseases. Surveillance of invasive species is therefore essential to assess the risks for mosquito-borne diseases and to prepare for a disease outbreak. The conventional strategies for controlling *Ae. albopictus* are based on reduction of breeding sites and using larvicides such as temephos and *Bti* in natural and/or peridomestic breeding sites [55]. If treatment with larvicides fails and in emergency situations, space spraying with pyrethroids or organophosphates can reduce the density of adult mosquitoes [55]. Alternative strategies consist of biological control (e.g., the use of larvivorous organisms or bioinsecticides), reduction of human-to-vector contact with insect repellents and insecticide-treated materials and genetic control (e.g., releasing factory-produced sterile insects or genetically modified mosquitoes that are unable to transmit diseases to humans). Unfortunately, few studies have shown effective, sustainable control of the *Aedes* mosquitoes with these methods [5]. Meanwhile, biological control, using copepod in genus *Mesocyclops* has allowed eliminating immature stage of *Ae. aegypti* in water storage containers in Vietnam [56]. Recent data showed that all *Ae. aegypti* and *Ae. albopictus* samples collected in Cameroon and Libreville (Gabon) were susceptible to *Bti* and temephos, and both species were fully susceptible to deltamethrin, except in Yaoundé, where the *Ae. albopictus* population had a mortality rate of about 80%, strongly suggesting resistance. WHO bioassays on adult mosquitoes showed resistance to dichloro-diphenyl trichlorethane (DDT) in one *Ae. aegypti* population in Gabon and two *Ae. albopictus* populations in Cameroon and suspected resistance to DDT in an *Ae. albopictus* sample from another site in Cameroon [57]. Vector surveillance and enhanced disease surveillance will enable early detection of cases and prompt implementation of control measures.

## Review conclusion

We have reviewed the current spread of the invasive species *Ae. albopictus* in central Africa, its larval ecology and its impact on the resident species, *Ae. aegypti*, and have explored the possible implication of *Ae. albopictus* in emerging or re-emerging arbovirus diseases. Various studies conducted in the region indicate that establishment and expansion of *Ae. albopictus* populations were facilitated by its ecological plasticity and by its ability to outcompete the indigenous species *Ae. aegypti. Ae. albopictus* thus found an environment similar to its native one, suggesting competition between this and native species. This invasive species is an efficient epidemic viral vector rather than a simple pest. The fact that central Africa has many potentially suitable niches for *Ae. albopictus*, as described in this review, and the presence of several endemic arboviruses of medical and veterinary importance could increase the risk for transmission of arboviruses such as DENV, CHIKV and ZIKV in central Africa. We have therefore reported measures for assessing the risk for mosquito-borne diseases and for preparing to control disease outbreaks.
